# Translating Molecular Approaches to Oligodendrocyte-Mediated Neurological Circuit Modulation

**DOI:** 10.3390/brainsci14070648

**Published:** 2024-06-27

**Authors:** Jingwei Song, Aybike Saglam, J. Bradley Zuchero, Vivek P. Buch

**Affiliations:** 1 Medical Scientist Training Program, School of Medicine, Stanford University, Stanford, CA 94305, USA; songjw@stanford.edu; 2 Department of Neurosurgery, Stanford University, Stanford, CA 94305, USA; saglama@stanford.edu (A.S.); bzuchero@stanford.edu (J.B.Z.)

**Keywords:** adaptive myelination, CNS biomarkers, deep brain stimulation, myelin plasticity, myelination, neural circuits, neurological gene therapy, neuropsychiatric disorders, oligodendrocyte precursor cells (OPCs), oligodendrocytes, synaptic modulation

## Abstract

The central nervous system (CNS) exhibits remarkable adaptability throughout life, enabled by intricate interactions between neurons and glial cells, in particular, oligodendrocytes (OLs) and oligodendrocyte precursor cells (OPCs). This adaptability is pivotal for learning and memory, with OLs and OPCs playing a crucial role in neural circuit development, synaptic modulation, and myelination dynamics. Myelination by OLs not only supports axonal conduction but also undergoes adaptive modifications in response to neuronal activity, which is vital for cognitive processing and memory functions. This review discusses how these cellular interactions and myelin dynamics are implicated in various neurocircuit diseases and disorders such as epilepsy, gliomas, and psychiatric conditions, focusing on how maladaptive changes contribute to disease pathology and influence clinical outcomes. It also covers the potential for new diagnostics and therapeutic approaches, including pharmacological strategies and emerging biomarkers in oligodendrocyte functions and myelination processes. The evidence supports a fundamental role for myelin plasticity and oligodendrocyte functionality in synchronizing neural activity and high-level cognitive functions, offering promising avenues for targeted interventions in CNS disorders.

## 1. Introduction

The central nervous system undergoes extensive physiological changes throughout life. Despite the majority of neurons reaching terminal differentiation early in life, the CNS has remarkable adaptability essential for learning and memory. Emerging evidence suggests that this adaptability is not strictly controlled by the neurons themselves, but that neuronal–glial interactions also influence neural circuit formation, function, and remodeling [1,2,3]. Among diverse glial populations, cells of the oligodendrocyte lineage, including oligodendrocytes (OLs) and oligodendrocyte precursor cells (OPCs), are critical for this adaptability. 

**Figure 1:**
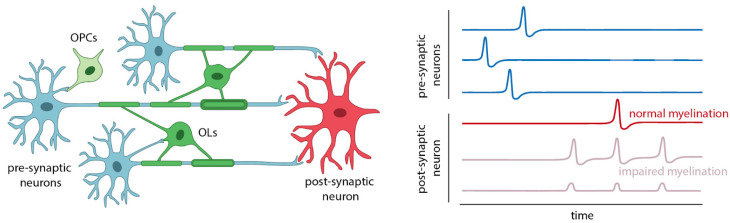
Oligodendrocytes, key to brain adaptability, produce myelin that speeds up signal propagation. Myelin’s size and morphology influence signal timing and synchronization, achieved through two interconnected processes: the formation of new myelin sheaths and the modification of existing ones. Impaired myelin dynamics result in asynchronous, spontaneous, and/or subthreshold activity in downstream neural pathways.

OLs and OPCs are interconnected with neural circuit development, differentiation, and remodeling via molecular and structural mechanisms (Figure 1). Molecularly, OLs and OPCs express receptors for neurotransmitters and reciprocally modulate synaptic transmission. Calcium dynamics in OPCs are responsive to both behaviorally correlated neuronal activity and direct neurotransmitter exposure [4]. Reciprocally, synaptic transmission, axonal excitability, and synchronous neuronal activity are modulated by OLs and OPCs [5,6]. Structurally, OLs and OPCs modulate neuronal circuits via myelination. Myelin is formed by OLs in the CNS, whereby compact lipid-rich lamellae are wrapped around axons for proper axonal conduction [7]. Although it was long thought that myelin was relatively inert after its formation, we now know that OLs dynamically modify myelin via changes in internode length, internode spacing, and sheath thickness; these are the adaptive mechanisms essential for the modulation of conduction velocity and circuit dynamics [8,9,10,11]. These mechanisms are orchestrated by several context-dependent cell biological processes, including extracellular cues, cell adhesion dynamics, cytoskeletal organization, and exocytosis for membrane expansion [11,12,13,14,15,16,17,18,19]. Further, OLs myelinate multiple axons in the CNS, which could provide an opportunity to integrate multinodal information processing into coordinated modifications of different neural circuits at once [20,21]. 

Accordingly, OLs/OPCs and myelin dynamics are implicated in memory acquisition and learning [22,23,24,25]. Adaptive changes in myelin sheath length and thickness occur in response to experience or experimentally induced neuronal activity [9,24,25,26,27]. Collective molecular evidence for the role of myelin plasticity in neural circuitry has been reviewed elsewhere [28]. Clinical observations based on EEG recordings from epileptic patients also provide insights into white matter functioning in higher-level cognition. In a recent study, intracranial recordings in humans performing a cognitive task showed a significant role of white matter communicability in the preparation of goal-directed cognitive tasks [29]. Proactive control, a term that describes the cognitive control mode in which prior information prepares the brain to be sensitive to goal-directed stimuli, was shown to be dependent on white matter communicability. These findings correlate observations in the development of network controllability in the perinatal period . Further, dynamic axonal conduction velocity and associated molecular mechanisms have critical physiological roles in synaptic transmission, temporal accuracy in neuronal activity, and high-level cognition and experience-directed circuit remodeling [25,30,31,32,33]. A recent report further revealed reciprocal modulations among reward-seeking behavior, oligodendrogenesis, and myelin plasticity [34].

The molecular mechanisms of adaptive myelination are likely fundamental to CNS functions such as information processing, storage, and retrieval [35,36]. These mechanisms remain an area of active investigation. In this review, we present the current understanding of OLs, OPCs, and myelination disease and disorders of the nervous system and discuss the potential for relevant future diagnostics and therapeutics.

## 2. OLs, OPCs, and Myelin in Neural Circuit Disorders

### 2.1. Epilepsy

Myelin dynamics and OL functionality play critical roles in the modulation of neural circuitry and are key contributors to the pathophysiology of epilepsy. Epilepsy represents the aberrant hyperexcitability of neuronal networks that propagate locally or globally in the brain. The mechanisms of ictogenesis and propagation involve diverse nervous system cell types. For example, electrical activity can propagate through gap junctions mediated by glial cell types including astrocytes and oligodendrocytes [37,38,39]. In epilepsy, myelin plasticity significantly influences the orchestration of neural network dynamics by modulating the speed and synchrony of signal transmission along axons. Disruptions in OL functionality and myelin integrity are implicated in epilepsy, where maladaptive myelination triggered by seizures contributes to increased seizure burden [40]. Additionally, impaired OL function disrupts potassium buffering, which can increase seizure likelihood [41]. For example, the thalamus is a critical area for the initiation and spread of generalized epilepsy. Molecular mechanisms that transition thalamic neurons from tonic to burst firing are linked to the precision of thalamocortical circuit synchrony which is impaired in the setting of myelin damage [42,43,44]. 

Microglia are additionally involved in interactions among neurons, myelin, and OLs/OPCs. Microglial gain or loss of function is associated with various myelin states, suggesting a regulatory role in maintaining myelin integrity [45,46,47,48,49,50]. Microglial activation in the setting of chemotherapy-induced toxicity leads to neurotoxic astrocytic responses, a loss of OLs and OPCs, and a reduction in myelin abundance and thickness [45,51]. In these experimental models, reduced BDNF-TrkB signaling was observed. Treatment with TrkB agonists or microglia depletion altogether restored DNF expression and myelination. Dysregulated TGFβF1 signaling in mature OLs in the setting of microglial activation was suggested as a mechanistic component of hypermyelination. Single-cell transcriptomic analyses further revealed OL clusters with elevated expression of SERPINA3 and C4B, which are markers linked to uncontrolled seizures in humans and risks for other nervous system diseases and disorders [46,52,53,54,55]. 

Cytokine IL-33 is also involved in microglial modulation of myelination and neural circuits [56]. IL-33 mediates the fine-tuning of the excitation/inhibition (E/I) balance and synaptic plasticity via multiple mechanisms, including the stimulation of neurons in the hippocampus and the remodeling of microglial synapses in corticothalamic circuits and the spinal cord [57,58,59,60]. Conditional deletion of IL-33 in astrocytes or its cognate receptor on microglia has been linked with an increase in excitatory synapses within the corticothalamic circuit [60]. This alteration correlates with the emergence of spontaneous epileptiform activities and a heightened susceptibility to seizures by early adulthood [58,61]. In addition, IL-33-deficient mice display failed OL maturation and impaired myelin structure, with behavioral phenotypes that include anxiety and impaired social cognition [62,63]. 

Taken together, these findings demonstrate that white matter connectivity mediated by OLs, OPCs, myelin, microglia, and intercellular molecular mediators serves as a basis for the synchronization of neural activity. Alterations in these dynamic processes can underlie E/I imbalances, ictogenesis, and the propagation of aberrant neural activity in epilepsy.

### 2.2. Glioma

OLs and OPCs are involved in the structural and functional connectivity between glioma tissue and surrounding native tissue. In gliomas, malignant cells form electrically active networks via synapses among themselves and with other neurons [64,65]. Electrical connectivity has functional roles in tumor proliferation, as demonstrated by studies where optogenetic stimulation of the optic nerve pathway accelerated glioma growth in mice and a reduction in optic nerve activity hindered glioma development [66]. Similar effects were observed in the olfactory bulb, where sensory experiences and neuronal activity influenced glioma growth, with interventions reducing tumor progression [67]. 

Malignant neuron–glioma synapses exploit mechanisms of adaptive plasticity typically seen in healthy neuronal synapses [68]. Activity-regulated secretion of BDNF increases both the number and strength of malignant synapses via enhanced α-amino-3-hydroxy-5-methyl-4-isoxazolepropionic acid receptor (AMPAR) trafficking to the postsynaptic membrane [51]. Interestingly, in gliomas, most AMPARs are calcium-permeable compared to the typical calcium-impermeable AMPARs in normal neurons due to aberrant processing of the GluA2 mRNA [69,70,71]. Studies highlight the role of AMPAR-mediated neuron-to-glioma synapses in aiding glioma invasion and expansion in the brain, with such synapses at the invasive tumor edge promoting tumor proliferation [68,72]. 

Network synchrony in gliomas is facilitated by cellular coupling among glial cells, leading to synchronous calcium wave formations that support tumor proliferation. Pharmacological blockage of such synchrony led to reduced glioma proliferation in mouse models [64,68,73]. Synchronous activity is attributed to enhanced tumor survival and growth. Studies that reversed neuronal activity induced autonomous rhythmic calcium oscillations in “hub” glioma cells, which in turn influenced neural circuit adaptability and signaling pathways [74]. Disruption of the Kca3.1 channel diminished such synchrony, decreasing tumor viability and growth, thereby extending survival in animal models, suggesting the potential of HCa3.1-inhibiting drugs as a novel treatment strategy [74]. Further, thrombospondin-1 (THBS1), a calcium-signaling-associated glycoprotein and astrocyte-derived signal for synaptogenesis during development, is implicated in glioblastoma progression, particularly influencing neural circuit remodeling through its expression in various cells within the tumor microenvironment [75]. 

Finally, OPCs often serve as the cell of origin for high-grade gliomas [72,73,76]. The dysregulated mechanics of myelin plasticity and paracrine signaling mediated by cells of the OL lineage also influence the glioma microenvironment, which may influence response to therapy [72]. 

### 2.3. Psychiatric Disorders

Interactions between microglia and OLs/OPCs in psychiatric disorders are poorly understood, but current understandings present intriguing therapeutic implications [77]. Stressful experiences have been shown to significantly affect oligodendrogenesis and myelination, especially in the prefrontal cortex (PFC) [8]. Experimental models demonstrate that stress leads to a reduction in myelin and OL-associated proteins [78]. These dynamics are in the setting of a larger framework wherein stress and psychoactive substances affect broad classes of glial cell types, especially in the prefrontal cortex [79]. The framework for understanding myelination abnormalities in psychiatric disorders so far implicates OLs/OPCs in regional hyper/hypomyelination and a loss of axonal metabolic support, which lead to altered connectivity and axonal degeneration, which impair higher-order cognitive functions [80]. 

Genetic changes in psychiatric disorders notably implicate OL-, OPC-, and myelin-related genes. In a study that identified 89 abnormally regulated genes in schizophrenia, 35 of them were involved in myelination, including genes encoding for myelin proteins (MAG, MAL, MBP, PLP, MOG, CNP), growth factors and receptors (ErbB3, NRG1, BDNF), transcription factors (SOX10, Olig1, Olig2), and other myelination-associated genes (transferrin, QKI, CLDN11) [81]. For example, CNP1, a myelin sheath protein, is crucial for maintaining normal axon function and survival [81,82]. Another gene encoding a myeline component, SOX10, is located in a major susceptibility locus for schizophrenia [83]. A loss of OL-derived erb-b2 receptor tyrosine kinase 4 (EBB4) signaling can result in OL arborization and subsequent impairments in myelin and dopaminergic function, which are involved in psychiatric disorders [84]. In the setting of depression, various molecular changes are observed. Microglia-derived factors that promote OPC proliferation and remyelination are downregulated in patients with depression [85]. Single-nucleus RNAseq of the dorsolateral PFC in depression identified the greatest number of differentially expressed genes in immature clusters of OPCs [86]. 

Beyond impairment at the genetic level, postmortem and live imaging studies further reveal significant white matter differences in bipolar disorder (BD) [87], schizophrenia [88,89,90], major depressive disorder (MDD) [91,92,93,94], and obsessive–compulsive disorder [95,96,97,98]. These changes are located throughout diverse brain regions associated with higher-order cognitive functioning, including frontal, insular, parietal, and other cortical regions. For example, postmortem examination has revealed significant oligodendrocyte reductions in the thalamic anterior principal and centromedian nuclei in bipolar disorder patients with psychotic episodes [99]. Schizophrenia patients show decreased oligodendrocyte density in cortical and thalamic regions [100,101]. 

Myelin changes can be ameliorated by administering antidepressants such as desvenlafaxine, indicating a potential therapeutic pathway through the modulation of myelination processes [102,103]. The therapeutic potential of antidepressants in the context of myelin repair is gaining attention. Antidepressants like desvenlafaxine have shown promise in improving myelin and OL-related proteins in animal models, suggesting their role in myelin repair mechanisms [102,103]. This opens up new avenues for exploring antidepressant therapies not just for symptomatic management in psychiatric disorders but also for addressing underlying myelin dysregulation.

Interestingly, recent developments have implicated impaired myelination in reward-seeking neurocircuitry, which underlies the pathophysiology of substance use and feeding disorders. Reduced white matter tracts connecting nuclei relevant to the reward system were observed in neuroimaging of adolescents with anorexia nervosa [104]. Furthermore, oligodendroglial lineage cells were found to respond to dopaminergic activity elicited in the reward pathway, and experimental blockade of oligodendrogenesis reduced rodent conditioning to opioids [34].

### 2.4. Neurodevelopment

Myelination is intricately linked with the stages of neurological development. During development, myelination begins spontaneously in the late fetal stages and continues into early adulthood, selectively ensheathing electrically active axons [105,106,107]. Phases of myelination can proceed independently of neuronal activity or can be adaptive to neuronal activity and experience [21,31,108,109]. Myelin-driven changes in neural conduction velocity, timing, and excitability can significantly influence developmental neurological functions [110,111]. Proper myelination is crucial for achieving developmental milestones in infancy and for the maturation of complex cognitive behaviors, especially in the PFC during adolescence [105,106,112]. The causal mechanistic understanding of adaptive myelination and calcium signaling has been subject to debate [4,108,113], limited by the availability of tools and challenged by the dynamic nature of these processes. Recent advances indicate the necessity of maintaining an intact actin cytoskeleton for proper myelin sheath formation and morphology [114].

Developmental alterations in myelination can generate vulnerabilities to neurological and psychiatric disorders. In cases of depression with a history of severe child abuse, there is a notable dysregulation in the transcriptional profiles of myelin genes coupled with a reduction in myelin thickness around small-caliber axons [115]. Glutamate signaling via distinct AMPAR subunits has been identified to regulate developmental OL function, which is also found to be casually linked to cases of SCZ [116,117]. Social isolation also reduces myelin-related transcripts and causes transcriptional and ultrastructural changes in PFC OLs [102,118]. Developmental cognitive delays can be caused by neonatal hypoxia that disrupts intracellular calcium levels, a process significantly influenced by GABAergic signaling. OPCs and myelinating OLs, which express calcium-permeable channels, are especially susceptible to ischemic conditions [119]. Enzymes such as sirtulin1 respond to calcium activity by regulating OL cytoskeleton and myelin dynamics and are possibly involved in hypoxia-induced developmental cognitive delays [120].

Adaptive tuning and maintenance of the balance between excitation and inhibition (E/I balance) are crucial for normal brain development and function to refine neural communication and processing. Notably, OPCs can receive synaptic inputs from excitatory or inhibitory neurons [121]. However, the potential role or contribution of these inputs in the E/I balance is not known. Alterations in the E/I balance have been postulated to underlie the pathogenesis of several developmental neuropsychiatric disorders, including autism spectrum disorder, schizophrenia, Down’s syndrome (DS), and attention deficit/hyperactivity disorder (ADHD) [122,123,124,125,126,127].

## 3. Diagnostics and Therapeutic Approaches

**Figure 2:**
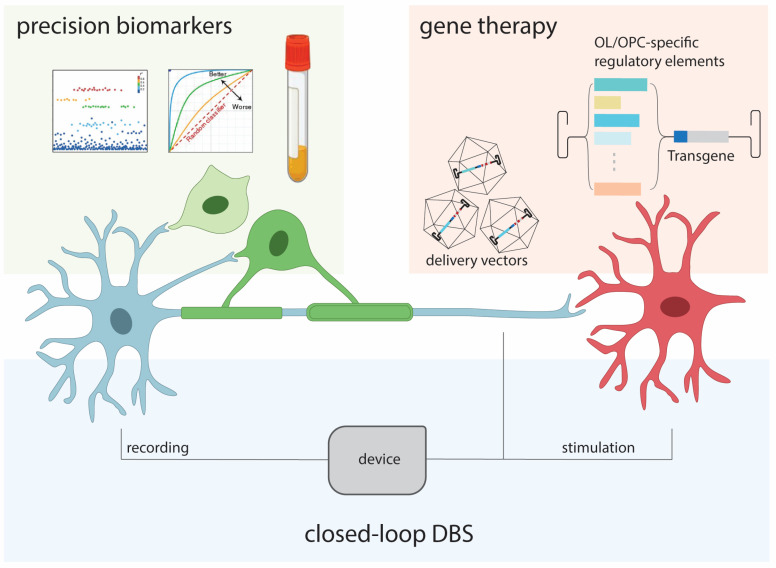
Summary of future diagnostic and therapeutic directions, wherein (i) clinical biomarkers including metabolomics, genomics, and neuroimaging may be used in combination with machine learning approaches to identify patients who would benefit from specific therapies, (ii) gene delivery modalities for targeted molecular perturbations, and (iii) closed-loop DBS for electrical restoration of neural connectivity implanted electrical devices.

The functional, structural, and molecular processes associated with OLs/OPCs and myelin, in conjunction with relevant microglial and neuronal biology, are extensively implicated in diverse neuropsychiatric diseases and disorders. These dynamics are candidates for future therapeutic strategies in the form of pharmacologic, biomarker-related, and neuromodulatory modalities (Figure 2).

The molecular mechanisms underpinning myelin dynamics and oligodendrocyte-mediated circuit modulation remain an area of active investigation. Beyond the aforementioned novel pharmacological possibilities, the current understanding of the molecular mechanisms underlying OLs and OPCs in disease states is insufficient to broadly permit new classes of pharmacological approaches. New pharmacological therapeutic modalities are bound to emerge as the basic biology underlying OLs/OPCs and myelination continues to mature. 

Interestingly, emerging biomarkers associated with OL dysfunction are providing crucial tools for understanding disease processes. The availability of multi-omics data, including genomics, transcriptomics, and epigenomics, all possible at the single-cell level, permits unprecedented characterization of molecular and cellular states associated with disease. For example, the discovery of factors associated with oligodendrogenesis within the cerebrospinal fluid (CSF) of young mice, in addition to their fluctuating levels during aging, can be leveraged to report global neuronal myelination states [105,127]. The implications of biomarker research extend to inflammatory diseases of the central nervous system, such as multiple sclerosis and pediatric demyelinating syndromes. Antibody-based biomarkers and myelin OL glycoprotein have now been developed for pediatric demyelinating syndromes [128]. In MS, OL-specific biomarkers can be leveraged to interpret disease severity. As the field advances, the integration of multi-omics biomarker research and data processing into clinical practice will be instrumental in refining diagnostic procedures and propelling the evolution of personalized treatment paradigms in neurological disorders. The feasibility of this approach will rely on the broader progress of precision medicine and will face universal challenges in bio-data collection, analysis, regulation, and integration into healthcare systems. 

Closed-loop deep brain stimulation (DBS) further offers opportunities to directly establish synchronous, or other temporally defined, activity in targeted neural circuits for functional improvement. DBS relies on intracranial electrodes to deliver electrical stimulation to anatomically defined regions. It represents a paradigmatic shift in surgical interventions of the human brain, whereby programmable electrical activity can directly modulate targeted gray and white matter regions for functional improvement. In this setting, closed-loop DBS combines real-time electrical recordings from the brain to analyze relevant disease states and gate electrical stimulation based on clinically determined parameters [129]. Such technology has been applied in the setting of episodic, fluctuating motor deficits in neuropathic pain, epilepsy, and Parkinson’s disease [130,131,132,133]. The field, to date, largely utilizes real-time recordings to determine electrical signatures of a functional brain state. However, theoretical applications extend to more nuanced restoration of neural circuit function. For example, for a hypothetical CNS nucleus whose axons innervate a destination nucleus, impaired myelination of these axons could result in impaired activity in the postsynaptic neurons (Figure 1). Closed-loops DBS, by way of real-time recording from the presynaptic cell bodies and targeted stimulation of the axons or the postsynaptic nucleus, could bypass areas of impaired myelination and directly establish electrical dynamics as a function of the recorded presynaptic activity. Such a concept would also benefit from the continued progress in high-density neural recordings and machine learning-aided interpretation of data [134].

DBS is limited in its indiscriminate stimulation of all cells and extracellular structures in the target region. In the future, direct molecular and cell-type-specific perturbations of OLs/OPCs are also possible, given recent advances in neurological gene therapy that offer the potential to genetically modify targeted cell types of the nervous system [135,136]. Chemical and genetic methods that selectively perturb non-neuronal cells of the nervous system have been shown to reverse behavioral deficits in rodent models [137,138,139,140,141]. Via minimally invasive modalities, astrocyte stimulation was directly shown to increase OLs, improve myelination, and improve behavioral outcomes in animals experiencing neuropathic pain [137]. The feasibility of targeted gene therapy toward OLs/OPCs in clinical settings is challenged by the ability to deliver transgenes to targeted cell types in the CNS and the identification of the therapeutic transgene within vector packaging limits. Possibilities in this realm will expand exponentially, with rapid progress in the fields of gene delivery to the CNS, transcriptional engineering for cell-type-specific transgene expression, and synthetic biology that provides diverse effectors for in vivo molecular modulation. For example, targeted gene delivery to OPCs may induce molecular reprogramming that promotes remyelination after axonal injury.

## 4. Concluding Remarks

In conclusion, the functional and structural roles of oligodendrocytes (OLs) and oligodendrocyte precursor cells (OPCs) within the central nervous system are fundamental to both the normal cognitive processes and the pathophysiology of various neuropsychiatric disorders. The dynamic interplay between myelination and neural activity not only supports cognitive functions such as learning and memory but also reveals the potential vulnerabilities where dysregulation can lead to neurological impairments. This understanding highlights the critical impact of OLs and OPCs in disease states, such as epilepsy, gliomas, and psychiatric disorders, pointing toward innovative diagnostic and therapeutic strategies that could significantly advance current treatment paradigms. By harnessing advances in molecular biology, genetics, and biomarker development, future research can enhance our ability to modulate these crucial cellular processes, potentially leading to novel and more effective treatments for a wide range of CNS disorders. This underscores the importance of continued research into the complex roles of OLs and OPCs, not only to broaden our basic scientific understanding but also to translate these findings into clinical interventions that can improve patient outcomes in neurological and psychiatric care.
